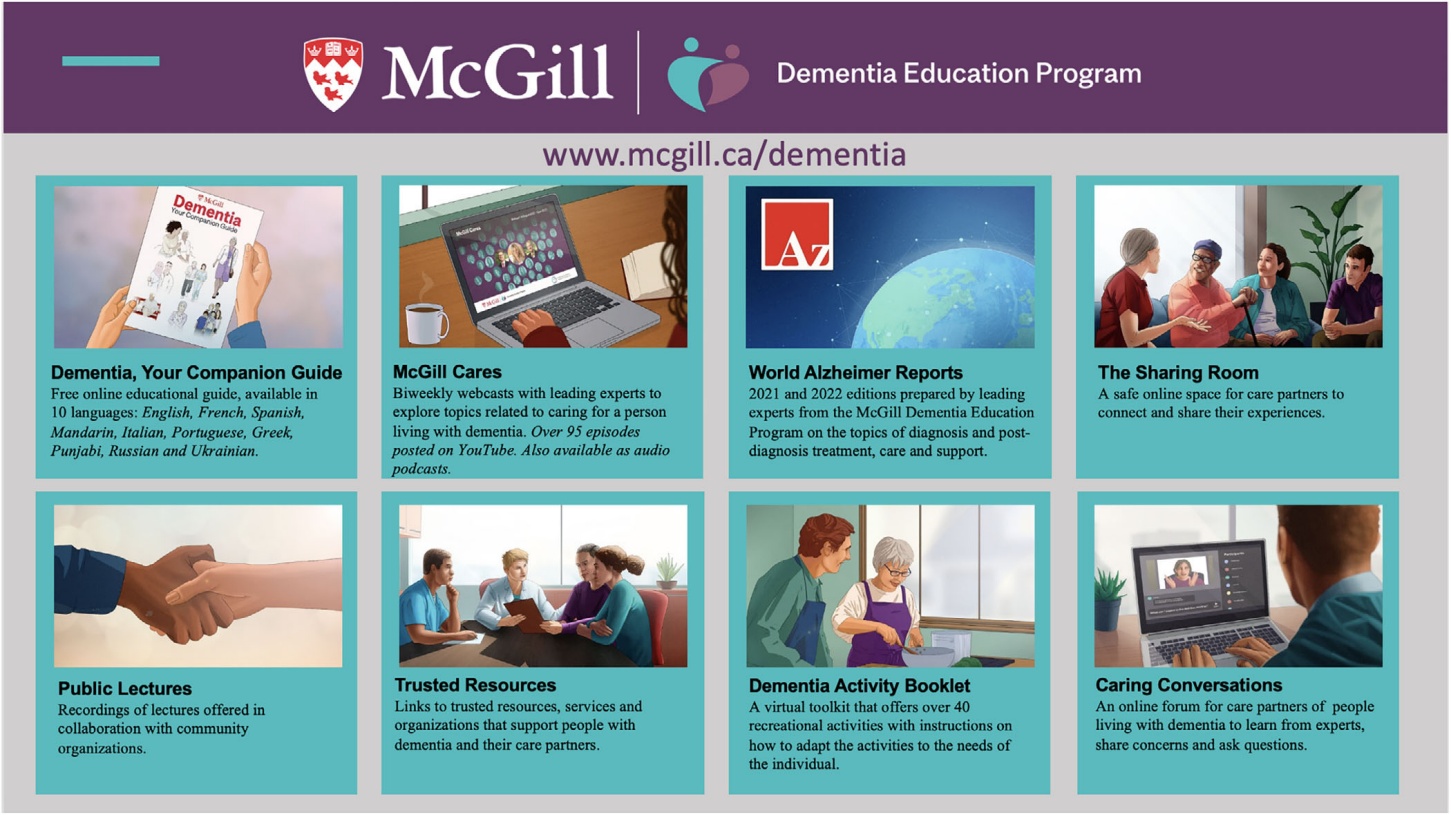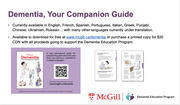# Bridging Academia and Community to Educate and Support Care Partners of People Living with Dementia

**DOI:** 10.1002/alz.083826

**Published:** 2025-01-09

**Authors:** Claire Webster, Serge Gauthier, Pedro Rosa‐Neto, José A Morais

**Affiliations:** ^1^ McGill University, Montreal, QC Canada; ^2^ Translational Neuroimaging Laboratory, The McGill University Research Centre for Studies in Aging, Montreal, QC Canada; ^3^ McGill University Research Centre for Studies in Aging, Montreal, QC Canada

## Abstract

**Background:**

Universities can play a very important role in providing post‐diagnosis dementia education and support to the community. The McGill University Dementia Education Program was founded in 2017 by Claire Webster, a former care partner and dementia care consultant with this aim. Mrs. Webster had no affiliation with the university prior to beginning the program other than being an annual guest lecturer on the topic of caregiving and dementia. Frustrated by the healthcare system, Mrs. Webster approached the university with an idea to introduce an innovative program centered on patient/care partner education using a multi‐disciplinary approach.

**Methods:**

The program offers a comprehensive range of free resources and cutting‐edge teaching and learning techniques, including simulation to educate and support persons living with dementia, family and informal care partners, healthcare professionals, medical students and the public at large; a dementia companion guide available in 10 different languages with many more currently in translation; over 100 educational webinars and podcasts known as “McGill Cares”; virtual support groups for care partners; 14 bilingual video capsules demonstrating leisure and recreational activities and an online education program currently under development for care partners.

McGill University has fully embraced the “person/patient centered care approach” into its medical curriculum by making in mandatory for all first‐year medical students to attend Claire Webster’s lecture on “Navigating the Journey of Caring for a Person Living with Dementia”.

**Results:**

Within only 5 years of its inception, the McGill University Dementia Education Program has received international recognition. The Academic and Medical Directors of the program (authors of this abstract) were selected by Alzheimer’s Disease International to write the 2021 and 2022 World Alzheimer’s Reports on the inter‐related topics of diagnosis and post‐diagnosis management. In 2023, Claire Webster was appointed to the Government of Canada’s Ministerial Advisory Board on Dementia.

**Conclusion:**

Universities can play a critical support role, bridging academia and community to provide service to society. They can institute community outreach programs that leverage the wealth of expertise within their institution, and they can integrate caregiver awareness and education into their medical school curriculum.